# The Emergence of a Novel Insertional Mutation in the BCR::ABL/p210 Oncogene in B-Cell Acute Lymphoblastic Leukemia (B-ALL) Correlates with the Development of Resistance to Several Tyrosine Kinase Inhibitors

**DOI:** 10.32607/actanaturae.27539

**Published:** 2025

**Authors:** K. V. Bogdanov, E. S. Kudryavtseva, Y. N. Lobacheva, O. V. Merzlikina, Y. V. Mirolyubova, R. A. Vlasik, R. Sh. Badaev, E. G. Lomaia

**Affiliations:** Almazov National Medical Research Centre, St. Petersburg, 197341 Russia

**Keywords:** B-ALL, BCR::ABL/p210, insertion, K294SPSQ, resistance to tyrosine kinase inhibitors

## Abstract

A patient with an immunophenotype characteristic of B-cell acute lymphoblastic leukemia (B-ALL) was found to carry the chromosomal translocation t(9;22)(q34;q11), or Philadelphia (Ph) chromosome and less common variant of the chimeric oncogene BCR::ABL/p210. No additional mutations in the BCR::ABL gene, including point mutations, insertions, or deletions, were identified in the disease onset characterized by elevated blast cell (77.6%) and leukocyte (48×109/L) counts. Ph+ALL-2012m chemotherapy with imatinib (600 mg) and two consolidation phases resulted in complete hematologic remission and a profound molecular response. However, six months later, the patient had relapsed (blasts: 15%, BCR::ABL/p210: 105%). Three weeks after the initiation of dasatinib therapy (100 mg), the number of blasts had decreased to 4.8%, while the expression level of BCR::ABL/p210 had dropped to 11.8%. Sanger sequencing identified two mutations in the BCR::ABL oncogene; namely, the point mutation F317L and a new insertion of nine nucleotides previously not detected. In the latter case, the amino acid lysine at position 294 was replaced by four new amino acid residues: K294SPSQ. Therapy with bosutinib and inotuzumab led to the disappearance of one leukemia clone with the F317L mutation, but the presence of another clone carrying a nine-nucleotide insertion was observed. The switch to ponatinib+blinatumomab chemotherapy was effective, resulting in the disappearance of the insertion. Allogeneic hematopoietic stem cell transplantation (allo-HSCT) from an available HLA-matched unrelated donor resulted in complete clinical and hematologic remission, including a complete molecular response. Six months after allo-HSCT, minimal residual disease monitoring showed maintenance of complete remission.

## INTRODUCTION

**Fig. 1:**
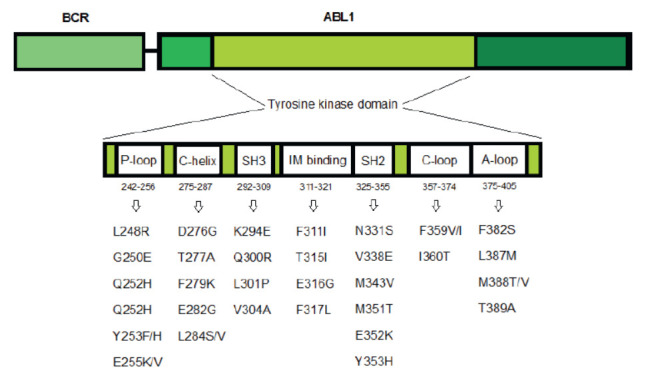
BCR::ABL tyrosine kinase domain mutations identified in patients with Ph-positive leukemia

Acute B-lymphoblastic leukemia (B-ALL) is a clonal proliferative disease of the blood system caused by genetic abnormalities arising in B-cell precursor cells. The disease is more common in children and less so in adults. Among adult B-ALL patients, some mutations are rare ( < 3%), namely: t(v;11q23)/MLL or KMT2A, including t(4;11)(q21;q23)/KMT2A-AF4, t(1;19)(q23;p13)/E2A-PBX1 (TCF3-PBX1), t(5;14) (q31;q32)/IL3-IGH, t(12;21)(p13;q22)/TEL-AML1 (ETV6-RUNX1), and C-MYC gene rearrangement mutations: t(8;14)(q24;q32), t(8;22)(q24;q11), or t(2;8) (p22;q23). In contrast, the Philadelphia chromosome (Ph chromosome) is a frequent mutation (up to 30%) among adult B-ALL patients. It results from reciprocal translocation t(9;22)(q34;q11), whose product is the chimeric BCR::ABL oncogene. Moreover, a variant of Ph-like B-ALL is usually identified among adult patients (≥ 20%). It is characterized by a molecular gene expression profile typical of Ph-positive B-ALL but different by the absence of the chromosomal translocation t(9;22)(q34;q11) and the predominance of a high frequency of deletions in the IKZF1 gene [1]. Most of these mutations correlate with a poor prognosis, except for some chromosomal translocations: in particular t(1;19)(q23;p13)/E2A-PBX1 and t(12;21) (p13;q22)/TEL-AML1. These chromosomal mutations are associated with an intermediate and favorable prognosis, respectively. In addition to correlation with poor prognosis in B-ALL patients, the chromosomal translocation t(9;22)(q34;q11) leads to an accumulation of the BCR::ABL oncoprotein, with constitutive tyrosine kinase activity. The increased ability of BCR::ABL to phosphorylate target proteins causes a transformation of hematopoietic stem cells, resulting in the alteration of multiple signaling pathways that enhance their survival and proliferation [2, 3, 4]. Different fusion products can be detected depending on the localization of the breakpoint in the BCR gene or alternative splicing of BCR-ABL mRNA. The most common ones are the BCR::ABL isoforms: e1a2 (p190), e13a2, and e14a2 (both p210). Meanwhile, most B-ALL patients (77%) tend to express BCR::ABL/p190 while a smaller proportion of patients (20%) express BCR::ABL/p210, and the remainder (3%) co-express BCR::ABL/p190 and BCR::ABL/p210 [5]. Despite the generally unfavorable prognosis for Ph-positive B-ALL, the prognosis is even worse for BCR::ABL/p210 mutation carriers compared to that for BCR::ABL/p190 mutation carriers. The advent of imatinib (IM), a tyrosine kinase inhibitor (TKI) suppressing the BCR::ABL tyrosine kinase activity, has significantly improved the hematological, cytogenetic, and molecular genetic characteristics of Ph-positive patients [6]. However, the majority of Ph+ B-ALL patients often develop resistance to IM via both the BCR::ABL-dependent and BCR-ABL-independent mechanisms [7, 8, 9, 10, 11, 12]. To overcome this resistance, second- (nilotinib, dasatinib, bosutinib), third- (ponatinib), and fourth-generation (asciminib) TKIs have been developed and introduced into clinical practice [13, 14]. The BCR::ABL-dependent factors contributing to resistance to TKIs include the mutations arising in the BCR::ABL gene that encodes the tyrosine kinase domain, including point mutations, insertions and deletions. The mentioned abnormalities can be detected both at the disease onset and during treatment. These mutations are rare (≤ 12%) at the disease onset, but their detection may increase during TKI treatment and contribute to the emergence of resistance. More than 100 point mutations in the BCR::ABL oncogene are currently known and have been previously described for both B-ALL and chronic myeloid leukemia (CML) [15, 16, 17]. These mutations affect different regions of the BCR::ABL kinase domain. Among those (1) the phosphate-binding P-loop (P-loop); (2) the C-helix site responsible for allosteric regulation; (3) the ATP/IM binding site; (4) the catalytic site (SH2 contact, SH3 contact, C-loop); and (5) the activation loop (A-loop) (Fig. 1).

Mutations are most commonly found in two regions of the kinase domain, the P-loop and ATP/IM binding site. The most common of those is the T315I (C>T) point mutation. It substitutes threonine for isoleucine, causing resistance to four different TKIs namely imatinib, dasatinib, nilotinib, and bosutinib [18]. Among the insertional mutations in the BCR::ABL kinase domain in B-ALL patients, insertions of two to twelve amino acid residues, usually localized between positions I293 and K294, as well as K294 and H295, are more frequently detected. In both cases, the structure of the SH3 contact site, which constitutes the tyrosine kinase domain of the BCR::ABL oncoprotein, is disrupted. It leads to the development of resistance to imatinib [19, 20]. Finally, among the deletions of the BCR::ABL oncogene in B-ALL patients, a Δ184–274 mutation has been described, as being associated with the loss of 90 amino acid residues. In particular, this disruption affects the P-loop region of the BCR::ABL tyrosine kinase domain, which correlates with resistance to TKIs, including ponatinib. [21]. The present study has revealed an increased expression of the BCR::ABL/p210 oncogene and two mutations in the BCR::ABL kinase domain in a patient with B-ALL (Ph+) after dasatinib therapy. The first one, the F317L point mutation, is well-known; and the second mutation, a new insertion of nine nucleotides, has not been described previously. This insertion results in the substitution of a lysine at position K294 for four amino acid residues of SPSQ, which is part of the SH3-contact site of the tyrosine kinase domain of BCR::ABL.

## EXPERIMENTAL

**Patient and samples**

**Table 1:** Main characteristics of the patient at the onset of B-ALL

Male	Peripheral blood						Bone marrow (blasts, %)
	hemoglobin, g/L	WBC, ×109/L	platelets, ×109/L	lymphocytes, %	monocytes, %	blasts, %	
Patient	101	48	30	15	3	67	77.6
Healthy person	130–160	4–9	150–400	19–37	3–11	0	0.1–1.1

A 42-year-old man was admitted to the Almazov National Medical Research Centre with a complain of pain in the knee joint and fever up to 38°C. According to the clinical analysis (Table 1), the patient had a high white blood cell count (48 × 109/L), as well as an increased blast count in the peripheral blood (67%) and bone marrow (77.6%). Immunophenotyping revealed a population of blast cells with the B-lineage phenotype: CD34+CD19+cytCD79a+CD10+CD38+sCD22+cytIgM- HLADR-CD13+MPO-CD33-CD117-. Hence, the patient was diagnosed with B-cell acute lymphoblastic leukemia (variant B II) with co-expression of the CD13+ myeloid marker. In addition, no involvement of the central nervous system was detected, since all the lumbar punctures showed the absence of leukemia cells in the cerebrospinal fluid.

**Cytogenetic analysis**

Preparation of chromosome spreads and subsequent chromosome differential staining were performed according to the assay described previously [22]. The karyotype pathology was interpreted by analyzing 20 mitoses by standard karyotyping and/or 200 interphase nuclei after fluorescence in situ hybridization (FISH).

**RNA isolation, reverse transcription (RT),** **and quantitative real-time PCR (qPCR)**

After the isolation of total RNA from 2.5 mL of peripheral blood and elution in 30 μL of RNase-free buffer, reverse transcription was performed using a standard set of reagents according to the manufacturer’s protocol (AmpliSense, Russia). Qualitative determination of the BCR::ABL fusion transcript variant was performed using microchip PCR and a 5× qPCRmix-HS reagent kit (Eurogen, Russia) as described previously [23]. Quantification of the BCR::ABL/p210 oncogene was performed using qPCR and the Leucosis Quantum M-bcr-FRT PCR kit (AmpliSens, Russia). After PCR, the amount (%) of BCR::ABL/p210 mRNA was calculated according to the standard formula: the number of BCR::ABL/p210 copies was divided by the number of ABL copies and multiplied by 100.

**DNA isolation**

Genomic DNA was isolated from 0.2 mL of peripheral blood using a QIAamp DNA Mini Kit (Qiagen, USA) according to the manufacturer’s protocol. DNA was eluted in 50 μL of the AE buffer (10 mM Tris-Cl, 0.5 mM EDTA, pH 9.0).

**Screening for BCR::ABL tyrosine** **kinase domain mutations**

**Table 2:** Specific oligonucleotides for PCR and direct sequencing of BCR::ABL

Primer	First round of PCR	Second round of PCR	Sequencing
Forward (5’-3’)	ACTCGTGTGTGAAACTCCAGACT	AGGACGAGTATGCGCTGAAG	AGGACGAGTATGCGCTGAAG
Reverse (5’-3’)	CGAGGTTTTGTGCAGTGAGC	CGAGGTTTTGTGCAGTGAGC	CGAGGTTTTGTGCAGTGAGC

The mutational analysis of BCR::ABL tyrosine kinase by Sanger sequencing was performed both after cDNA amplification preceding RT, and after amplification of a specific region of genomic DNA. In the first case, standard nested PCR was performed using oligonucleotides as well as amplification and thermocycling conditions as described previously [24]. In the second case, we performed long-range PCR using two-round PCR and a BioMaster LR HS-PCR reagent kit (Biolabmix, Russia) to identify mutations in the BCR::ABL oncogene in genomic DNA. Amplification and thermocycling conditions did not differ for both rounds of PCR, except for the use of specific oligonucleotides selected through the NCBI system (Table 2). Each sample for the second round of PCR contained 1× PCR buffer, 2.5 mM of each dNTP, 10 pM forward and reverse primers, 3 μL of the amplification product (after the first round of PCR), and 5 units of Encyclo Taq polymerase (Evrogen, Russia). The thermocycling steps included the initial holding at 95°C for 10 min, followed by 50 cycles: 95°C for 15 s and 60°C for 1 min. The size of the amplification product after the second round of PCR was 293 bp. To determine the mutational status of BCR::ABL, Sanger sequencing was performed using an ABI PRISM 3500 genetic analyzer (Applied Biosystems, USA).

## RESULTS

**Mutation analysis at the onset of B-ALL**

**Fig. 2:**
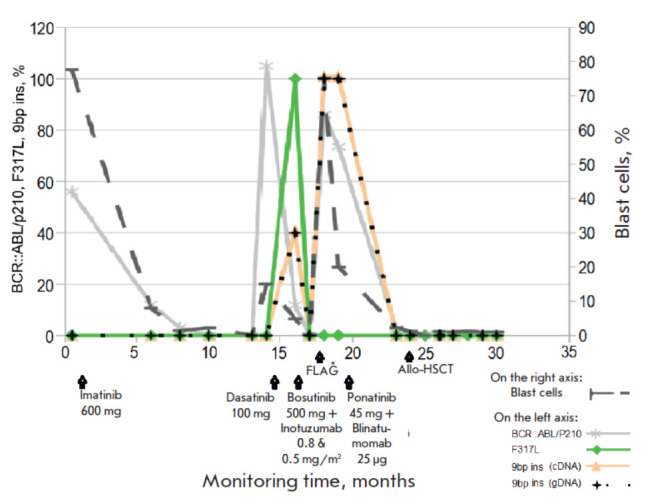
Analysis of biomarkers at the onset of B-ALL and during treatment. FLAG* therapy includes FLAG + venetoclax (100 mg) + asciminib (400 mg). The number (%) of BCR::ABL/210 oncogene transcript mRNA (mutation) was estimated relative to the ABL reference gene (wild type). The number (%) of blast cells was determined relative to the total number of all nucleated cells in the bone marrow

According to standard karyotyping, an abnormal karyotype was identified in the patient’s bone marrow cells: {t(9;22)(q34;q11) [15], 46XY [5]}. A Ph chromosome was found in 15 of 20 mitoses (75%). Furthermore, the BCR::ABL oncogene was detected in 120 of the 200 interphase nuclei viewed using FISH. According to the quantitative PCR data, increased expression of the BCR::ABL/p210 oncogene was found in peripheral blood, its level not exceeding 56% (Fig. 2). No additional mutations were found in the BCR::ABL gene encoding the tyrosine kinase domain.

**Therapy and monitoring of minimal residual disease (MRD)**

**Fig. 3:**
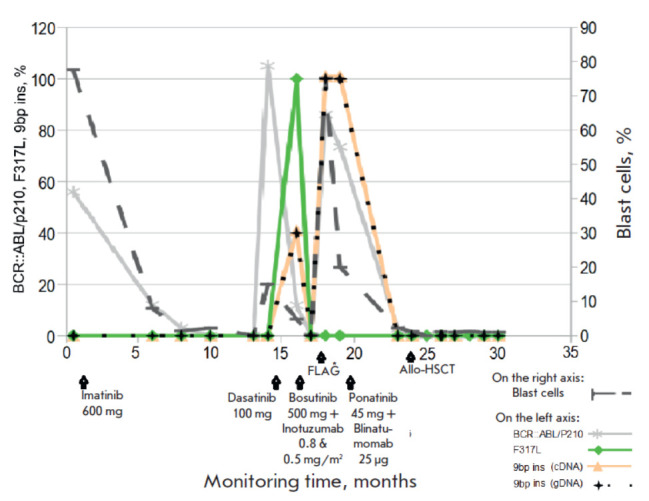
The sequence of a novel insertion in the tyrosine kinase domain of BCR::ABL found in a B-ALL patient. (A) At the onset of B-ALL: no mutation was detected. (B) B-ALL relapse: insertion (K294SPSQ) was detected

After securing informed consent, treatment was initiated according to the Ph+ALL-2012m protocol, in combination with imatinib (600 mg) [25]. After 8 months of therapy that included two induction and three consolidation phases, complete hematologic remission and a profound molecular response (BCR::ABL/p210: 0.002%) were noted. However, six months later, the patient had a relapse (blasts: 15%, BCR::ABL/p210: 105%). After three weeks of therapy (100 mg dasatinib + 20 mg dexamethasone), the number of blasts was reduced to 4.8% and the level of BCR::ABL/p210 decreased to 11.8%. Sanger sequencing analysis showed a T-to-C nucleotide substitution at position 949 (NM_005157) of the ABL gene, resulting in the F317L point mutation. Meanwhile, a novel nine-nucleotide insertion (GCCCTTCCC) at a position between 1073 and 1074 (NM_005157) of the ABL gene was found. The latter mutation caused a lysine substitution at position K294 for four amino acid residues; namely, serine-proline-serine-glutamine (K294SPSQ), which preceded histidine at position H295 (Fig. 3).

Once the results of the BCR::ABL mutational status were available, dasatinib was replaced with bosutinib. Treatment with bosutinib (500 mg) + dexamethasone (40 mg), in combination with two courses of inotuzumab therapy (0.8 and 0.5 mg/m2), was performed. The patient showed a decrease in the blast count to 0.2% and the BCR::ABL/p210 mRNA expression level, to 0.069%. However, 1.5 months after completion of this therapy, an increase in the blast count to 75.2% and an increase in the BCR::ABL/p210 level to 86% were found in the patient’s bone marrow. Sanger sequencing showed the absence of the F317L mutation but presence of a nine-nucleotide insertion. After FLAG+venetoclax (100 mg) + asciminib (400 mg) therapy, the blast count in the bone marrow decreased to 20% (BCR::ABL/p210: 73.5%). Sequencing again confirmed the presence of the insertion. Change of therapy to ponatinib (45 mg) + blinatumomab (28 μg) resulted in the disappearance of the leukemia clone with the insertion, which correlated with a complete molecular response and complete clinical and hematologic remission. One month after chemotherapy, the patient underwent allo-HSCT from an available HLA-matched unrelated donor. Monitoring of the BCR::ABL/p210 oncogene expression and its mutational status confirmed the absence of any molecular genetic abnormalities during the last six months after allo-HSCT. The patient is currently in MRD-negative remission.

## DISCUSSION

This article describes a rare clinical case of Ph-positive B-ALL with a chimeric variant of the BCR::ABL oncogene, typical of CML and characterized by a number of features. First, no mutations in the tyrosine kinase domain of BCR::ABL were detected in the patient at the onset of B-ALL (Ph+, BCR::ABL/p210+). Second, the development of resistance to dasatinib correlated with the detection of two tumor clones. One of them carried a point mutation F317L, while the other one carried a novel nine-nucleotide insertion accompanied by a four amino acid substitution of lysine at position K294 namely serine-proline-serine-glutamine (K294SPSQ). The insertion did not result in a reading frame shift. Third, after the emergence of resistance to dasatinib, the patient was found to be refractory to bosutinib and asciminib. Meanwhile, the disappearance of one leukemia clone carrying the F317L mutation and the presence of another clone carrying a nine-nucleotide insertion were noted. Only a therapy switch to ponatinib+blinatumomab resulted in complete eradication of the clone carrying the insertion. According to earlier literature data, the F317L point mutation localizes in the region of the BCR::ABL kinase domain that is responsible for imatinib binding (IM binding site) [26]. In addition, carriers of this mutation are resistant to imatinib and dasatinib but sensitive to bosutinib [18]. In our case, treatment (bosutinib+dexamethasone) in combination with two courses of inotuzumab therapy resulted in the disappearance of the F317L mutation. However, the nine-nucleotide insertion remained. Meanwhile, the patient had an elevated expression level of the BCR::ABL/p210 oncogene and increased number of tumor cells, which correlated with leukemia progression (Fig. 2). Interestingly, the nine-nucleotide insertion associated with the replacement of lysine by four new amino acid residues is located in the SH3 contact site of the tyrosine kinase domain of BCR::ABL. Mutations leading to resistance to TKIs are known to occur most frequently in this region as well as in the P-loop region [27]. According to earlier studies, the SH3 contact site is required for the autoinhibition of ABL tyrosine kinase in normal cells [28]. Mutations, and especially insertions at this site impair the aforementioned function. In addition, according to recent studies, mutations in the BCR::ABL gene leading to the modification of contact sites (SH2 and SH3), along with destabilization of specific tertiary structures of the protein and large-scale conformational changes, are considered to be additional mechanisms for the emergence of resistance to TKI [29]. In our case, the emergence of a nine-nucleotide insertion in the SH3 contact site of the tyrosine kinase domain of BCR::ABL led to the discovery of a new SPSQ motif. It appears that serine phosphorylation of this motif through the involvement of one of the serine/threonine kinases, such as Dyrk1A, may contribute to the progression of the leukemia. Dyrk1A was previously found to be involved in the phosphorylation of several targeting proteins, including Amph1, which also possesses an SPSQ motif [30]. Interestingly, increased expression of Dyrk1A was recently detected in B-ALL (BCR::ABL/p190+) [31]. Activation of the JAK/STAT-signaling pathway was shown to promote the proliferation of leukemia cells overexpressing Dyrk1A. The same investigators, using a mouse model of B-ALL (BCR::ABL/p190+), suggested that Dyrk1A may be involved in the regulation of BCR::ABL expression. In particular, an artificially induced heterozygous Dyrk1A deficiency in these mice may help prevent leukemic cell survival, thereby promoting the normalization of hematopoiesis.

## CONCLUSIONS

In this study, a patient with B-ALL (BCR::ABL/p210+) developed resistance to several TKIs (dasatinib, busutinib, and asciminib), as well as to polychemotherapy including the FLAG regimen. This was accompanied by an increase in BCR::ABL/p210+ expression and the emergence of two leukemia clones, one of which included a point mutation (F317L) and the other one, a nine-nucleotide insertion (GCCCTTCCC) involving the substitution of lysine at position K294 for four new amino acid residues (K294SPSQ). In the described case of B-ALL, the elevated resistance to TKI may be due to an increase in the level of serine phosphorylation in a new SPSQ motif involving the serine/ threonine kinase DyrkA1. This may lead to the activation of the JAK/STAT signaling pathway, ultimately enhancing cell proliferation and supporting leukemogenesis.

## Abbreviations

B-ALL: B-cell acute lymphoblastic leukemia
CML: chronic myeloid leukemia
TKI: tyrosine kinase inhibitor
IM: imatinib
allo-HSCT: allogeneic hematopoietic stem cell transplantation
